# Enhanced medical image segmentation using optimized bidirectional LSTM and dolphin partner optimizer

**DOI:** 10.1371/journal.pone.0342592

**Published:** 2026-02-20

**Authors:** Afnan M. Alhassan, Nouf I. Altmami

**Affiliations:** Department of Computer Science, College of Computing and Information Technology, Shaqra University, Shaqra, Saudi Arabia; Khalifa University, UNITED ARAB EMIRATES

## Abstract

Medical imaging has become a standard in diagnosing and treating how organs and tissues operate. In earlier systems, machine learning approaches were used for segmentation over a long period. Thus, processing and analyzing these medical images are highly important for clinical diagnosis. Deep learning-based image segmentation has attracted a lot of interest in recent years. This paper introduces the Optimized Bidirectional Long-Short Term Memory (OBi-LSTM) segmented technique for medical image classification. The OBi-LSTM classifier contains the sequencing data in both directions, backward and forward. It is utilized to simultaneously be aware of the most significant spatial placements, channels, and scales. Using Dolphin Partner Optimizer (DPO), the weight and bias parameters of the Bi-LSTM classifier are tuned for each cell. Since these variables are shared across the entire sequence, the network can maintain effective representation while requiring fewer unique weight parameters and hidden neurons, reducing overall computational complexity. OBi-LSTM classification outperforms state-of-the-art techniques in the MRI segmented test, demonstrating the proposed approach’s usefulness and strong explanatory power. The proposed OBi-LSTM model achieves a high dice similarity coefficient of 94.05%, a Jaccard resemblance index of 88.77%, and an accuracy ratio of 93.05% compared to other existing models. The proposed OBi-LSTM + DPO model significantly enhances medical image segmentation performance, particularly in boundary delineation and feature extraction.

## 1. Introduction

In several applications of computer-aided diagnostics, medical image classification is crucial. Researchers are drawn to use new medical image-processing algorithms because of the significant investments and growth of medical imaging modalities, including Positron Emission Tomography (PET), Computed Tomography (CT), Magnetic Resonance Imaging (MRI), and X-ray. Due to its ability to automatically or semi-automatically extract the Region of Interest (RoI), image segmentation is regarded as the most crucial step in medical imaging. When used in medical applications for boundary identification, tumour identification, and mass detection, it separates an image into sections according to a given description, such as segmenting bodily parts and tissues [1].

Clustering algorithms may be used for segmenting by identifying the global properties of the image to expertly distinguish the ROI from the background, since classification divides the image into coherent areas. There are several methods for clustering, including mean shifting categorization, contentious categorization, hierarchy clustering, and K-means clustering. Additionally, fuzzy set and neutrosophic set theories become crucial in the segmentation process to address uncertainty in the medical images, owing to the irregular and fuzzy boundaries in most medical images. As a result, it is possible to use the Fuzzy C-Means (FCM) [2], and Neutrosophic C-Means (NCM) clustering is introduced to enhance various segmentation approaches. The clustering process requires the number of clusters and their centroids to be determined beforehand [3].

Additionally, the gradients and brightness data are utilized for image classification. It is possible to apply various categorization techniques, including some based on boundaries, like the deformation model, and others that are region-based techniques, like regional combining, region expanding, and active contour [4]. However, most medical images have poor borders, noise, and inhomogeneous intensity, necessitating complicated methods. [5].Intelligent/advanced techniques for medical image classification have become a hotspot, leading to hybrid approaches for effective classification based on the border and ROI. These techniques include graph-based algorithms like graph cut classification, which iteratively separates the image’s object and background, utilizing specified classification restrictions by picking seed points indicating object and background pixels.

A graph-based technique segments images depending on the maximal flow/minimum cut between origin and sink vertices in direction networks. Graph Cut (GC) algorithms are efficient in medical image classification. GC approaches need time-consuming participatory selection of object/background seeds. To overcome this problem, Kernel Graph Cut (KGC), a completely automatic GC process, was created [6]. Skin lesion segmentation is one of the most difficult applications of medical image segmentation. The blurry lesion boundaries and their irregular features, skin lines, hairs, air bubbles, multicoloured areas inside, and the poor contrast between the lesion and the surrounding skin sections are diverse lesions that may be seen in dermoscopic images, however [7]. Such artefacts increased the need for the Neutrosophic Set (NS), which is crucial for removing ambiguity throughout segmentation and developing an automatic skin lesion Computer-Aided Diagnosis System (CAD). Existing medical image segmentation approaches still have a way to go before they overcome all the obstacles deep learning introduces. Failing segmentation in low-contrast and very diverse medical pictures is a common problem with traditional convolutional models like U-Net and its variations, since these models have confined receptive fields, making capturing long-range relationships difficult. Though they enhance global context modelling, transformer-based systems are impractical for use in real-time due to the high computing costs they impose. Standard LSTM models are unidirectional and can not fully use bidirectional spatial correlations in medical pictures, even though recurrent networks such as LSTMs may keep sequential dependencies. Because they can not dynamically adjust to complicated loss landscapes, traditional optimization methods like Adam and RMSprop often converge to less-than-ideal solutions. A cutting-edge segmentation method is required to address these drawbacks, combining adaptive optimization with bidirectional sequential modelling for optimal accuracy and computing efficiency.

Deep learning (DL) networks have improved efficiency and attained great correctness levels on various well-known databases during the last several years, helping to design novel image segmentation models [8]. Semantic segmentation and instance segmentation are two main categories for image segmentation techniques. Semantic segmentation may be seen as a classification issue for pixels. Every pixel in the image is assigned to a certain class using this segmented process. Each RoI in the input image is found and identified via example separation.

The main contribution of the paper is

- This study offers a hybrid skin lesion segmentation system by DPO and Bi-LSTM segmentation to provide an effective and unsupervised skin lesion segmentation solution.
- Then, a unique channel attention module is proposed to emphasize the most important feature channels and adaptively adjust channel-wise feature responses.
- Additionally, suggest a scale attention module that implicitly emphasizes the most important feature mappings across various scales so that the CNN can adjust to an object’s size.
- The proposed Bi-LSTM system greatly increased the average segmentation accuracy, according to extensive trials on skin lesion segmentation from ISIC 2018 and multi-class segmentation of fetal MRI.

The rest of the sections are organized as follows. The skin lesion segmentation and deadly MRI segmentation approaches are covered in detail in Section 2, along with numerous related research studies. The structure of the many approaches employed in the proposed method is explained in Section 3. Section 4 contains a detailed overview of the experimental findings. Section 5 concludes generally with recommendations for further development.

## 2. Related work

Many articles have spent a lot of time researching different segmentation techniques and attributes. The issue is complicated and lacks a universally applicable solution due to the wide and ever-increasing variety of objects involved, variations in their image characteristics, different medical imaging modalities, and associated changes in signal homogeneity, variability, and noise for each object. Here we take a look at the most popular methods for medical picture segmentation and discuss their capabilities, basic advantages, and possible downsides.

Over the last decade, applications like computer-aided diagnosis have made great use of Deep Neural Networks (DNNs) for medical picture segmentation. On the other hand, properly segmenting very complicated, low-contrast pictures of tissues and organs remains a challenge. Zhang et al. [9] created a novel model called Star-shaped Window Transformer Reinforced U-Net (SWTRU) by combining the transformer, which is powerful in capturing global contexts, with the U-Net network, which excels at picture segmentation. By reducing network parameters, the proposed method has the potential to increase computation efficiency.

For effective feature extraction processing, Wu et al. [10] created DI-Unet, which stands for Dimensional Interactive (DI) self-attention. Inputting high-resolution pictures and collecting cross-dimensional data before calculating attention levels might lead to efficient minimization of model calculations. The huge benefit of DI-Unet has been shown by extensive testing across several datasets. When compared to other methods, the suggested method outperforms them on classification tasks involving large datasets. Objects’ long-distance dependence cannot be faithfully replicated by the responsive field, unfortunately.

Scalable operational finite difference Bayesian Neural Networks (BNNs) using Gaussian Processes were proposed by Chen et al. [11] for medical picture segmentation. Using content-aware reassembly of features (CARAFE) as an up-sequencing controller, this technique develops a content-aware UNet segmentation network that retrieves conceptual data from input feature maps. It sets its loss goal using a modified version of fELBO, which places emphasis on the Kullback-Leibler Divergence (KLD) term. The suggested BNN system is capable of reasoning about predictions and enables efficient training with only a single forward pass. But choosing relevant prior distributions and fitting appropriate posterior distributions in a high-dimensional weight space is challenging, which limits BNN’s practical applicability.

As a means of achieving better segmentation with fewer variables, Han et al. [12] suggested ConvUNeXt. Despite using 20% less variables than the standard UNet, the suggested model outperforms it when it comes to data segmentation, regardless of the amount of data present. This was shown in tests conducted on different datasets. Elements such as medical picture contrast, heterogeneity, and boundary sharpness put limits on current convolutional algorithms, making it impossible for them to correctly locate boundaries.

Using deep learning models, An and Liu [13] suggested an interactive self-attention module. It may enable the feature map to collect global data and realize effective extraction of medical picture feature information. This fixes the problem where the medical pictures don’t line up well with the deep learning network’s design. This study employs a deep learning model with many layers of border-perceiving methods to segment images. The ideas given before formed the basis for this algorithm’s development. However, without sufficient foundational data, its placement choices are limited.

Khosravanian et al. [14] introduced a Fuzzy Local Intensity Clustering (FLIC) that combines fuzzy clustering with a level set technique. This approach was introduced to reduce the impact of noise contamination and variations in intensity. The FLIC technique may provide fully automated and contemporaneous segmentation and bias correction via local intensity clustering and beginning contour selection using the fuzzy approach. The regional concentration of intensity makes this feasible.

A Saliency-Guided Morphology-aware U-Net (SMU-Net) was reported by Ning et al. [15] for the purpose of lesion segmentation in Breast Ultrasound (BUS) images. The components of the SMU-Net are a main network, an additional middle stream, and an auxiliary network. Particularly, the first stage need to propose creating saliency maps that take into account both the low-level and high-level picture structures of the backdrop and foreground, respectively.

Medical image segmentation that is more accurate and understandable was suggested by Gu et al. [16] using a Convolutional Neural Network (CNN) architecture and a Comprehensive Attention Convolutional Neural Network (CA-Net). This approach takes into account the most important spatial locations, channels, and scales all at once. CNN is only useful for medical decisions because to its low interpretability. Complex circumstances with large location, shape, and size variability in the classification aim will continue to be a difficulty. In contrast to U-Net, the suggested CA-Net significantly improved the average segmentation Dice score for the skin lesion, the placenta, and the fetal brain from 87.77% to 92.08% in the comprehensive tests conducted on skin lesion segmentation from ISIC 2018 and multi-class segmentation of fetal MRI.

With the use of a deep guiding network, Yin et al. [17] were able to partition the biological picture. The suggested model includes a guided image filter module that may be used to rebuild the structure’s content using the guiding picture. Training can be completed in its entirety using the suggested method, and inferences may be made quickly. Classifying vessels, segmenting optic discs, and classifying cups all need extensive testing at this stage. Nevertheless, there are jobs where the structural information cannot be completely retrieved, such segmenting retinal vessels. This is due to the fact that the vessels themselves are just curved lines, which cannot be identified even after several convolutions. This holds true for a number of other jobs as well.

Enhanced transformer (MAXFormer) was proposed by Zhiwei Liang et al. [18] for merging of multi-scale features and multi-attention in medical picture segmentation. Our Transformer module streamlines the self-attention process by disentangling its components into external attention and local-global attention. The parallel architecture of local-global attention makes it an efficient and linearly complex alternative to self-attention by allowing local-global spatial interactions. The low-frequency global information is handled by the global attention branch, while the high-frequency local information is handled by the local attention branch. We have also developed the Refined Fused Connection module to successfully fuse the feature outputs from each encoder block with the decoder output, therefore reducing the loss of spatial information that occurs during downsampling.

Bayesian Modeling for Medical Image Segmentation with Interpretable Generalizability was suggested by Gao et al. [19]. The Hierarchical Hybrid Transformer (H2Former) was suggested by He et al. [20] for the purpose of medical image segmentation. By integrating the greatest aspects of hierarchical hybrid vision Transformers (H2Former), CNNs, and multi-scale channel attention, the author is able to effectively segment medical pictures. These characteristics allow the model to make good use of sparse medical data. This method beats previous Transformer, CNN, and hybrid methods on three 2D and two 3D medical image segmentation tasks, as shown by the experimental results. It also maintains computational efficiency for the model parameters, FLOPs, and inference time. H2Former outperforms TransUNet on the KVASIR-SEG dataset by 2.29% with 30.77% parameters and 59.23% FLOPs, resulting in a better IoU score.

In order to segment 3D medical images, Cheng Chen et al. [21] presented a modification of the Modality-agnostic Segment Anything Model (MA-SAM). A subset of weight increments is updated using the parameter-efficient fine-tuning strategy, which primarily keeps SAM’s pre-trained weights, at the beginning of this approach. Our method enables the pre-trained 2D backbone to extract 3D information from incoming data by injecting a series of 3D adapters into the image encoder’s transformer blocks. Using 10 public datasets that include CT, MRI, and surgical video data, the author has rigorously validated our method on four medical picture segmentation tasks. As an example, our approach outperforms nnU-Net in Dice’s CT multi-organ segmentation by 0.9%, MRI prostate segmentation by 2.6%, and surgical scene segmentation by 9.9%—all without any prompt—which is rather remarkable.

For medical image segmentation, Jing Zhang et al. [22] discussed the Swin Transformer enhanced U-Net with Cross-Layer Feature Enhancement. For the purpose of cross-layer feature learning, a novel module called Cross-Layer Feature Enhancement (CLFE) is proposed. You may highlight certain locations by using a Spatial and Channel Squeeze & Excitation module. Last but not least, convolutional neural networks (CNNs) are utilized to train the fused features of the CLFE module. This allows for more accurate semantic segmentation by retrieving low-level data and localizing local characteristics. Using the open-source Synapse and ISIC 2018 datasets, our suggested ST-Unet achieved experimental values of 78.86 dice and 0.9243 recall, surpassing the performance of the majority of current medical image segmentation methods.

A medical picture segmentation approach that uses cloud computing and multi-feature interaction and fusion was presented by Xianyu He et al. [23]. In order to overcome limitations in local computer capability, this method use cloud computing to examine many medical images. Furthermore, it improves segmentation accuracy using a novel interactive fusion attention module and a CNN-Transformer hybrid for local feature extraction. The proposed technique has been validated on many medical imaging datasets, and experimental results demonstrate its efficacy and advancement.

For the purpose of analyzing medical images, Maciej A. Mazurowski et al. [24] introduced the Segment Anything Model. SAM’s IoU for single-prompt performance ranges from 0.1135 for spine MRI to 0.8650 for hip X-ray, with large variations between datasets and tasks. It seems that segmenting organs on CT scans and other objects with clear boundaries and clear instructions works better than segmenting brain tumors in other situations. As opposed to point prompts, SAM performs much better when given box cues. RITM, SimpleClick, and FocalClick are similar solutions, however when it comes to establishing a single point prompt, SAM always comes out on top. Repeated delivery of multiple-point cues only slightly improves SAM’s performance, even while other techniques surpass its point-based performance.

The Salp Swarm Algorithm Improved Reptile Search Algorithm (RSA-SSA) for Medical Image Segmentation was studied by Laith Abualigah et al. [25]. With the use of Otsu’s variance class function, the proposed RSA-SSA calculated the best threshold values for each level. A performance metric for the proposed method may be established by determining the ftness function, structural similarity index, peak signal-to-noise ratio, and Friedman ranking test. Several COVID-19 benchmark images show that the proposed RSA-SSA achieves its goals. Previous optimization approaches disclosed in the literature have relied on metaheuristics; the proposed RSA-SSA outperformed them.

The efficient CNN and Transformer supplementary network for medical picture segmentation was investigated by Feiniu Yuan et al. [26]. We start by building two encoders using Swin Transformers and Residual CNNs, respectively, to provide additional features in the Transformer domain and the CNN domain. Following that, we propose a Cross-domain Fusion Block (CFB) to effectively merge these supplemental attributes using crosswise concatenation. In order to gather data on two-way attention, we compute the correlation between features in the CNN and Transformer domains and apply channel attention to the self-attention features produced by Transformers. To improve the representational capacities of features, we offer a Feature Complementary Module (FCM) that integrates cross-domain fusion, feature correlation, and dual attention. Finally, we improved the representation of long-range relationships by developing a Swin Transformer decoder. To further extract geographical details, contextual semantics, and long-range information, the author recommends using skip links between the characteristics that the Transformer decoded and the complementing features.

In their study on medical picture segmentation, Francis H. Shajin et al. [27] examined the Sailfish optimizer using Levy flight, chaotic, and opposition-based multi-level thresholding. Using the optimum multi-level threshold based on Otsu’s and Kapur’s entropy techniques, brain, lung, and abdominal images are segmented in this case. For optimal segmentation outcomes while processing medical pictures, we employ Levy flight sail fish optimizers (LFSFO), chaotic sail fish optimizers (CSFO), and opposite sail fish optimizers (OSFO) to refine the weight parameters of Otsu’s strategy and Kapur’s entropy. The proposed MLT-LFSFO-CSO-OSFO-MIS method achieves a reduced mean square error and surpasses three prior methods in terms of performance and accuracy.

Automated Knowledge Transfer (AKT) based on deep learning was proposed by Jinal Mistry for medical image segmentation. Finding the most relevant information from existing styles and applying it to the target project is what AKT does best, therefore it might outperform guiding engineering techniques. This article’s authors [28] utilize the HAM10000 dataset, which has 10,015 pictures, to apply segmentation and transfer-learning algorithms to dermatoscopic skin-lesion images. A number of pre-trained classification networks, including ResNet50, ResNet152, SqueezeNet1.1, and DenseNet121, as well as U-Net, ResUNet, and DeepLabV3+, have been tried as segmentation approaches. The most effective method was DeepLabV3+, which achieved 96.21 percent accuracy, 93.26 percent precision, and 93.26 percent recall.

As a consequence, you get a reliable answer that works in many contexts. This article describes the AKT program, which can segment medical images, and its speech skills. The focus of the conversation is on recent articles and topical concerns in the area. We provide a synopsis of the three most recent AKT-specific procedures—transfer newest, meta-today, and self-supervised modern-day in order to accomplish beneficial medical image segmentation. In its last section, the article suggests ways forward for research in this field. The conventional approaches are summarized in Table 1.

**Table 1:** Summary of Traditional Methods.

Author(s)	Method	Results	Advantages	Limitations
Zhang et al. [9]	Star-shaped Window Transformer Reinforced U-Net (SWTRU)	Improved segmentation performance with reduced network parameters	Enhances computing efficiency and captures global context using the Transformer	It may require extensive computational resources for training
Wu et al. [10]	Dimensional Interactive (DI) self-attention for DI-Unet	Better classification on large datasets, reduced model computations	Efficient feature extraction and reduced computations for high-resolution images	Limited ability to model long-distance dependencies
Chen et al. [11]	Bayesian Neural Network (BNN) with Gaussian Process (GP) and content-aware UNet	Enables effective training with a single forward pass	Improves feature reassembly using CARAFE and reduces computational complexity	Selecting prior distributions and fitting posterior distributions in high-dimensional space is challenging
Han et al. [12]	ConvUNeXt (optimized UNet)	20% fewer parameters than standard UNet while maintaining high segmentation accuracy	Reduces model complexity while preserving segmentation quality	Struggles with medical image contrast, heterogeneity, and boundary sharpness
An & Liu [13]	Interactive self-attention deep learning model	Improved global feature extraction in medical images	Enhances network adaptability to image data structures	Limited placement possibilities due to insufficient fundamental information
Khosravanian et al. [14]	Fuzzy Local Intensity Clustering (FLIC) with level set algorithm	Automated segmentation and bias adjustment	Effectively mitigates noise and intensity variations	Computationally intensive and sensitive to initialization parameters
Ning et al. [15]	Saliency-Guided Morphology-aware U-Net (SMU-Net)	Enhanced lesion segmentation in Breast Ultrasound (BUS) images	Integrates low- and high-level structural details for accurate segmentation	Requires extensive training data for optimal performance
Gu et al. [16]	Comprehensive Attention Convolutional Neural Network (CA-Net)	Increased Dice score (from 87.77% to 92.08%) in skin lesion, placenta, and fetal brain segmentation	Improves spatial, channel, and scale-based attention for precise segmentation	CNN’s interpretability issues limit its clinical applicability
Yin et al. [17]	Deep guiding network with guided image filter module	Improved vessel classification, optic disc segmentation, and cup categorization	Fast training and inference due to guided image filter module	Lost structure information is difficult to recover in tasks like retinal vessel segmentation

### 2.1. Inference

New segmentation difficulties are appearing due to the fast growth and expansion of medical image modality, and new approaches are being presented and studied to find solutions to these challenges. The most effective strategies incorporate several image/object attributes and data analysis methods. Even though tests provide more precise findings, the categorization process often becomes too difficult and time-consuming. It is necessary to develop accurate and quick segmentation algorithms based on deep learning that can be used in real-world CAD systems.

### 2.2. Research gap/limitations

The related work section is now presented with more critical and synthesized discussion following substantial revision. We have eliminated the arrangement of current models strictly in chronological order and instead classified earlier work into three categories of frameworks: CNN, hybrid RNN-CNN, and transformer’s models. Each category reveals the limitations of spatial-temporal modeling, which ultimately motivated our method, OBi-LSTM + DPO, that considers both optimization stability and representivity of contextual features.

## 3. Proposed methodology

In order to enhance medical image segmentation, the study presents an Optimized Bidirectional LSTM (OBi-LSTM) and the Dolphin Partner Optimizer (DPO). The proposed approach uses the DPO for adaptive tuning of parameters, which is a departure from traditional Bi-LSTM models, leading to accelerated convergence with improved boundary delineation. The OBi-LSTM structure, optimization, and segmented workflow will be addressed in further detail in subsequent sections.

**Fig 1:**
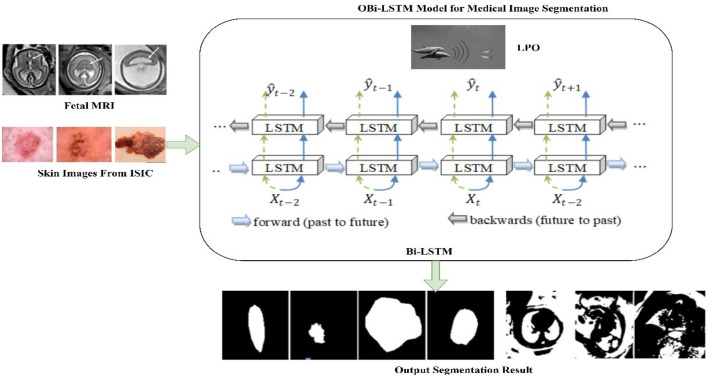
Optimized Bidirectional Long-Short Term Memory (OBi-LSTM) for skin lesion segmentation.

Optimized Bidirectional Long-Short Term Memory (OBi-LSTM) is proposed for classification, having better precise and understandable medical image classification, as illustrated in Fig 1. Any neural network may be made to contain sequence information in both forward and backward using the OBi-LSTM classifier. It is utilized to simultaneously be conscious of the most significant spatial placements, channels, and scales. The number of weights and hidden neurons in the network is decreased by computing the proposed OBi-LSTM classification, weight, and bias values in each cell using DPO. OBi-LSTM classifier outperforms such conventional edge techniques in the MRI segmentation challenge, demonstrating the efficiency of the proposed approach. Metrics like the Jaccard Similarity Index (JSI), Dice Similarity Coefficient (DSC), and reliability were utilized to evaluate the classification outcomes.

### 3.1. Bi-LSTM-based skin lesion segmentation

Fig 1 depicts the methods that will be used in the proposed study. The skin lesion categorization method that has been presented uses two methods to recover skin lesion areas from photos of impacted skin disease. The image of the hidden skin lesion is first segmented into blocks a size of nm, and then the gradient, intensity, and ridge method is used to obtain characteristics from individual blocks within the image. The process of extracting features produces feature vectors as its final product. Bi-LSTM model receives these feature vectors as an input, and the DPO technique is utilized to modify the hyperparameters of the Bi-LSTM model. This section presents a potential classification method and explains when it can effectively distinguish the foreground and background of the examined skin lesion. It incorporates the techniques of feature extraction, Bi-LSTM, and DPO altogether. Bi-LSTM is given the output vector that was generated by characteristics. In the latent skin lesion, segmentation aims to differentiate between the background and the foreground. The bi-LSTM approach was utilized to segment the latent skin lesion. The feature extraction module’s output is sent as a matrix to the Bi-LSTM [29], which serves as the input. The gradient vanishing issue may also be reduced using the DPO technique, which is utilized for weight updating in the Bi-LSTM model. The LSTM architecture comprises a forget gate, memory cell, input gate, and output gate, among the other fundamental elements. The output of the LSTM may be calculated by utilizing the equations (1–5), which are shown below,


$${\overset{―}{C}}^{t}=\text{tan}\omega \left(h_{lc}L^{t}+k_{\omega c}\omega^{t-\text{1}}+B_{c}\right)$$ (1)

$$B^{t}=\Sigma \left(k_{lb}L^{t}+k_{cb}C^{t-\text{1}}+B_{b}\right)$$ (2)

$$I^{t}=\Sigma \left(k_{lf}L^{t}+k_{cf}C^{t-\text{1}}+B_{I}\right)$$ (3)

$$C^{t}=F^{t}\theta C^{t-\text{1}}+B^{t}+{\overset{―}{C}}^{t}$$ (4)

$$D^{t}=\Sigma \left(K_{lo}L^{t}+k_{wo}\omega^{t-\text{1}}+k_{co}C^{t-\text{1}}+B_{o}\right)$$ (5)

where $\;{\overset{―}{C}}^{t}$, $B^{t}$, $F^{t}$, $C^{t}$and $D^{t}$ storage cell, input gate, output gate, present memory cell, and forget gate are all represented by this symbol. The $k_{l}$, $k_{\omega }$ represents the weight matrices for the LSTM $L^{t}$ and B identifies, and refers to the input and biased variables Σ and tanw and hyperbolic tangent functions, respectively, are referred to as the sigmoid and hyperbolic tangent functions. You may figure out the output of the LSTM in its final form by utilizing equation (6).


$$\omega^{t}=D^{t}\theta \text{tan}\omega \left(C^{t}\right)$$ (6)

LSTM can learn the time series information above, but Bi-LSTM can also learn the context information associated with time series. A backward LSTM network and a forward LSTM network are the two components that make up the Bi-LSTM system. $L^{\text{1}},L^{\text{2}},\ldots ,L^{t}$ are the input phases, and the result in both the backward and forward directions determined at every time ${\overset{\leftarrow }{\omega }}^{t}$ and ${\vec{\omega }}^{t}$ respectively. The output in its ultimate form then $y^{t}$ is arrived at by calculating both the forward and the backward output. The backward memory cell state and the forward memory cell state are, respectively, ${\overset{\leftarrow }{\overset{―}{C}}}^{t}$ and $C^{t}$, output gate is $\overset{\leftarrow }{D^{t}}$, and $\vec{D^{t}}$ input gate is $\overset{\leftarrow }{B^{t}}$, and $\vec{B^{t}}$, present memory cells are $\overset{\leftarrow }{C^{t}}$, and $\vec{C^{t}}$, and forget gates are $\overset{\leftarrow }{F^{t}}$, and $\vec{F^{t}}$. It can determine the ultimate output at time t using equation (7).


$$y^{t}=\left[\overset{\leftarrow }{C^{t}},\;\vec{C^{t}}\right]$$ (7)

where $\overset{\leftarrow }{C^{t}}$ represents the LSTM that operates in the reverse direction when it receives input, and the $\vec{C^{t}}$ represents the LSTM that operates in the forward direction and accepts the input. Utilizing DPO allows for the determination of the Bi-hyperparameters. LSTM’s [30]. The bilateral processing of OBi-LSTM makes it possible to understand relationships across entire spatial sequences and deals with contextual dependencies in both directions. This dual flow of input, when compared to any design that relies on unidirectional LSTM or CNN, offers a much higher ability to differentiate complex anatomical characteristics and subtle tissue differences, resulting in much higher accuracy in segmentation. Intensity features aid in differentiating between various kinds of tissue based on differences in pixel intensity, while gradient-based features improve edge recognition, which is critical for defining lesion borders. The ridge approach improves segmentation accuracy for complicated areas by capturing tubular structures and small features. The dice similarity coefficient and Jaccard index decrease when these traits are removed, suggesting they are significant according to the empirical data. Further validation of these extracted features’ contribution to segmentation accuracy is provided by Grad-CAM visualizations, which show that the model largely depends on them for decision-making.

### 3.2. Dolphin partner optimization based optimal hyperparameter value selection

Physicians have built a superior neural network model to analyze patients’ healthy skin and unhealthy skin states, and this improvement is utilized to improve the hyper-variables of Bi-LSTM. Dolphins utilize sound to navigate, locate food, and communicate with one another. They also cooperate to locate their prey. A dolphin will search for neighbours before choosing a friend to be his companion. He has a self-organizing team with all of his collaborators that now functions as a virtual dynamic team. Once each dolphin has established his team, he may interact with them to determine the best position and fitness level. The solution with the highest fitness will be chosen as the BiLSTM hyperparameter once the solution’s fitness has been determined. His teammates may recognize the superior dolphin after multiple iterations of this data interchange. The activity number is calculated accurately, and the greatest result is obtained when the fitness is at the lowest possible level. The performance function measures how much an individual is better than the community. When the Dolphin Partner Optimizer is used in a cooperative metaheuristic process, the learning rate, number of hidden units, batch size, dropout rate, and hyperparameters of Bi-LSTM are modified. Unlike the traditional optimizers like Adam or RMSprop, DPO converges faster, overfits less, and is more accurate, and is inspired by the social foraging behavior of dolphins to balance exploration and exploitation behavior. The fitness value shows how well a person performs. A person performs better when they are in good physical shape. The decision of whether a person will live or pass away is made depending on the fitness functional values. This study uses the Dolphin Partner Optimization (DPO) technique to get the best environments for the Bi-LSTM model’s hyperparameters. Modelling the partner selection and movement behaviours of dolphins in a dynamic environment, DPO balances exploration and exploitation, taking inspiration from the cooperative foraging behaviour of dolphins. Each possible solution, or dolphin, in a search space of hyperparameter candidates is assessed using a fitness function that quantifies the model’s performance, usually employing validation loss or accuracy. Dolphins navigate with adaptive position updates, which allow solutions to change depending on which partners are doing the best. This process continues until an ideal configuration is achieved via iterations.

**Fig 2:**
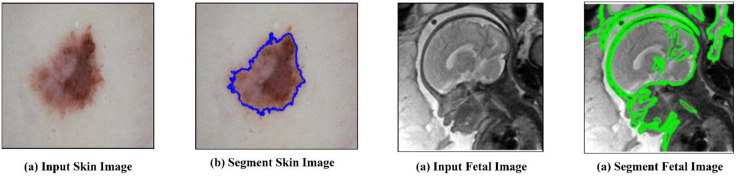
Implementation of images of the input skin image and fetal image with its output.

As a result, the DPO algorithm’s driving element, as illustrated in Fig 2, is the fitness functional. The current optimizing technique, such as PSO, shows its efficiency compared to GA; it may be a local optimum in high-dimensional space. However, DPO is quick-tempered, adapts well to various target functions, and offers the finest answers. The DPO is a method that examines all hyperparameters to assess their fitness and reliability. Numerous hyperparameters are available in this method to fulfil user requirements; these Bi-LSTM hyperparameters are originally filled in with the character “j”. J may have values ranging from 0 to 1000. Using this method, the DPO chooses its partner and creates a self-organizing team, which is a dynamic team at that point.

After building a team, each dolphin interacts with its teammates to improve Bi-posture LSTM’s and fitness. His partners understand the optimal hyperparameters after multiple data exchanges. According to DPO, this study collected the safer and certified virtual machines and then used a streamlined safety assessment to choose the most qualitative and safe virtual machine. To check for assaults in a virtual machine and provide a counteraction, the streamlined security analysis again identifies the characteristics. It will choose the most protected option with the least time needed depending on them. Many hyperparameters make up the Bi-LSTM. Each of these hyperparameters runs the Bi-LSTM, where x=0…. n, ‘n’ indicates the most hyperparameters possible in a Bi-LSTM that are made available to consumers for the conclusion of their procedure. The assumptions below are made using certain natural dolphin behavioural traits to create a novel optimizing method influenced by nature. Using a fitness function that maximized the Dice similarity coefficient while reducing loss and computational cost, key hyperparameters, including learning rate, number of LSTM units, dropout rate, and batch size, were tuned. The superiority of DPO may be shown by contrasting its search space and convergence behaviour with those of well-established optimizers such as Adam or Bayesian Optimization.

- The only things dolphins do in their search area are search, hyperparameters, or hunt sardine schools.
- Whenever dolphins pursue swarming sardines in the water or swimming with the best possible hyperparameter values, they exclusively engage in flip or rotational swimming.
- Every dolphin exclusively utilizes its sonar to interact with friends, transmit ultrasonic waves, identify echoes, and locate objects.
- Three situations may be used to categorize the entire search procedure: finding the targeted’ whereabouts, pursuing and approaching the objectives, and lastly, preying upon or determining the targets’ ideal classifier values.
- The dolphin may constantly switch between various swimming modes and search strategies according to its needs.

When looking for a hunt, dolphins first scan the whole area. They steadily raise the clicking production frequency as they come closer to the objective while also narrowing the search area. To regulate its capacity for investigation and utilization, the DPO algorithm utilizes a convergence factor (CF), which is the proportion of replies that occur the most often. This method alters the converging factor according to a function established for the converging rate. Following are the details for this feature,


$$P\left(l_{i}\right)=P_{\text{1}}+\left(\text{1}-P_{\text{1}}\right)\frac{l_{i}-\text{1}}{lN-\text{1}}$$ (8)

where $P\left(l_{i}\right)$ is a known possibility in phase i, $P_{\text{1}}$ is a first-step present possibility, $l_{i}$ is the most recent action amount, and lN is the amount of stages the algorithm needs to do to get the best result. Once the required repetitions have been reached or the ideal hyperparameter for BiLSTM has been identified, altering the dolphin’s location will be repeated. One sees the pseudocode for the DPO-based hyperparameters variable update in the algorithm.

### Algorithm 1. Pseudocode of DPO-based hyperparameter value Selection

**Input:**

$X\;\in \;R^{H\times W\times C}$ → Input medical image

$Y\;\in \;{\left\{\text{0},\text{1}\right\}}^{H\times W}$ → Ground truth segmentation mask

θ = {Wf, Wb, bf, bb} → Initial Bi-LSTM weights and biases

α, β  → Control parameters for DPO

**Output:**

$\hat{\text{Y}}\;\in \;[\text{0},\text{1}{]}^{H\times W}$ → Predicted segmentation mask

**Steps:**

*1. Normalize image:* $\;X\;\leftarrow \frac{\left(X\;-\;\mu \right)}{\sigma }$

*2. Convert X into sequential patches* $\left\{x^{\text{1}},\;x^{\text{2}},\;\ldots ,\;x_{T}\right\}$

*3. Initialize hidden states:* $\;h^{\text{0f}}\;=\;\text{0},\;h^{\text{0b}}\;=\;\text{0}$

*4. For* t = 1 to T do

*5.* $h_{{\text{t}}^{\text{f }}}=\;LSTM_{f\left(x_{t},h_{{\left\{\text{t}-\text{1}\right\}}^{\text{f }}};\;Wf,\;bf\right)}$

*6.* $h_{{\text{t}}^{\text{b}}}=\;LSTM_{b\left(x_{\left\{T-t+\text{1}\right\}},\;h_{{\left\{\text{t}-\text{1}\right\}}^{\text{b }}};\;\right)}Wb,\;bb$

*7. End for*

*8. Concatenate states:* $\;H_{t}=\;\left[\;h_{{\text{t}}^{\text{f }}}\left|h_{{\text{t}}^{\text{b}}}\right|\right]$

*9. Compute attention:* $\;\alpha_{t}=\;softmax\left(H_{t}W_{a}\right)$

*10. Context vector:* $\;C\;=\;\Sigma_{t}\alpha_{t}H_{t}$

*11. Initialize dolphin population* $\left\{\theta_{i}\right\}$

*12. For generation* g = 1 to G do

*13. Evaluate fitness* $f_{i}=\;L\left(Y,\hat{\text{Y}}\;\left(\theta_{i}\right)\right)$

*14. Update* $\theta_{i}=\;\theta_{i}+\;DPO_{Update\left(\theta_{i},\;f_{i},\;\alpha ,\;\beta \right)}$

*15. End for*

*16. Select optimal* $\theta *\;=\;argmin\left(f_{i}\right)$

*17. Predict* $\hat{\text{Y}}\;=\;sigmoid\left(CW_{o}+\;b_{o}\right)$

*18. Threshold:* $\;if\;\hat{\text{Y}}\;\geq \;\text{0}.\text{5}\;\to \;\text{1}\;else\;\text{0}$

*19. Compute Dice loss* $L=\text{1}-\left(\frac{\text{2}𝛴Y\hat{\text{Y}}}{\left(𝛴Y+𝛴\hat{\text{Y}}\right)}\right)$

*20. Return* $\hat{\text{Y}}$

The study optimized the Bi-LSTM hyperparameters using Dolphin Partner Optimization (DPO). These hyperparameters include learning rate, number of hidden units, dropout rate, and batch size. Instead of using backpropagation updates, which might be affected by starting circumstances, DPO can search globally for optimum hyperparameters without being stuck in local minima. This is in contrast to typical gradient-based optimizers like Adam. Using attention maps and feature significance visualization approaches, the OBi-LSTM model was made more interpretable. Grad-CAM was used to create heat maps that showed which parts of the input photos were the most important to help with segmentation choices. To further understand how the model analyzes medical pictures, SHAP (SHapley Additive Explanations) was used to quantify the influence of each feature on the model’s output. These explainability methodologies guarantee the model’s accuracy and transparency, which allow doctors to check its decision-making process. Techniques like Grad-CAM, SHAP, and attention maps are vital in medical image segmentation because they reveal how a model finds and processes important information. Reducing scepticism and promoting uptake, these explainability approaches enable physicians to analyze whether the model’s emphasis matches medical thinking. Even the most effective AI models may have trouble being approved by regulators and integrated into real-world healthcare settings if they aren’t transparent.

Unlike standard CNNs, which rely on local receptive fields, Bi-LSTM captures contextual relationships across entire feature maps, enhancing segmentation accuracy, especially in complex medical images with irregular shapes and textures. Moreover, Bi-LSTM mitigates the limitations of pure convolution-based models by incorporating bidirectional spatial awareness, improving boundary delineation. Empirical results demonstrate that integrating Bi-LSTM with convolutional layers outperforms standalone CNN-based architectures in handling structural continuity and contextual coherence, making it a viable choice for medical image segmentation.

## 4. Experimental results and discussion

The proposed framework is tested in this section using two examples: Dermoscopic images segment binary skin lesions. Fetal MRI segmentation uses several classes, which include the fetal brain and placenta. Implemented ablation experiments were used in both applications to confirm the usefulness of the proposed OBi-LSTM. It was evaluated to existing approaches like ConvUNeXt [12] and CA-Net [16] based on efficiency criteria including accuracy, the Jaccard Similarity Index(JSI), and the Dice Similarity Coefficient (DSC). The effect of various hyperparameter settings on segmentation accuracy was investigated using a sensitivity analysis. The influence on the Dice similarity coefficient (DSC), Jaccard index, and inference time was assessed by varying the learning rate (0.0001–0.01), number of Bi-LSTM units (32–256), dropout rate (0.1–0.5), and batch size (8–64). The results revealed that dropping the LSTM units below 64 resulted in a 3.4% loss in the Jaccard index because of limited feature extraction capability and that raising the learning rate beyond 0.001 generated instability, resulting in a 2.8% reduction in DSC. Results showed that a dropout rate of 0.3 was ideal, with a 1.9% performance hit when values were higher. In addition, the segmentation accuracy suffered along with the inference time when the batch size was more than 32. The OBi-LSTM model is trained to acquire spatial-temporal patterns based on the raw medical data, unlike the conventional machine learning algorithms that rely on features which are designed. It is more accurate at segmentation and efficient but more important than methods like FCM, K-means, and Graph Cut, due to its deep bidirectional form, which also makes it more adaptive to intensity variations and complex textures.

### 4.1. Dataset description

This study utilized a publicly available, fully anonymized dataset from the ISIC 2018: Skin Lesion Analysis Towards Melanoma Detection Challenge. The data were obtained from the International Skin Imaging Collaboration (ISIC) archive and contain no personally identifiable information. Therefore, ethical approval and informed consent were not required for this research.

The ISIC 2018’s publicly accessible training set, which includes 2594 images and their corresponding ground truth, to divide skin lesions. There was the adoption of a regular preparation pipeline to deal with the various sets of medical pictures. To obtain the same solution, all of the photos were downsized, normalized, and intensity-scaled. Such preprocessing ensures that the model itself is stable and allows the generalization of the model to other datasets of varying formats and acquisition parameter, such as prenatal MRI scans and ISIC skin pictures.

The database was divided randomly into 1816, 260, and 518 groups for learning, verification, and assessment purposes. The skin lesion identification database was originally between 720540 and 67084439 pixels in scale. Every image was downsized to 256 342 pixels and standardized using the average values and standard deviation. Random rotation with an angle in and 224 300 random cropping, vertical and horizontal flipping, and random rotation (−π/6, π/6) were implemented to improve the data. The dataset comprises 150 stacking with three perspectives of T2-weighted fetal MRI scans performed on 36 pregnant women during the second trimester using Single-shot Fast-Spin echo (SSFSE) with pixel sizes ranging from 0.74 to 1.58 mm and inter-slice spacing between 3 and 4 mm. The gestational age varied from 22 weeks up to 29 weeks. Spina bifida was found in 8 of the fetuses, but the remaining fetuses did not have any significant fetal abnormalities. All pregnant women had to be older than 18, and the hospital’s Research Ethics Committee had to give their blessing before using any data. The segments were then arbitrarily divided at the patient level, and 1050 patients were allocated to the learning group, 150 patients to the verification group, and 300 patients to the assessment group. The OBi-LSTM model addresses the issue of class imbalance by using weighted loss and oversampling of poorly represented classes of lesions. Further to ensure equal exposure in the training, data augmentation is used which involves flipping and rotation. The techniques are used to remove bias to dominant classes of structures; these techniques generate better segmentation of small or rare medical structures. The test set comprises 110 axials, 80 coronals, and 110 sagittal slices. As the underlying truth, an expert radiologist manually annotated the fetal brain and placenta. For concurrently identifying these two organs, a multi-class classification network was created, and each slice was scaled to 256 × 256 pixels. This correction increases the grammatical accuracy and is an understandable description of the preprocessing step used on the MRI dataset. To enhance the data, the x and y axes were randomly rotated and flipped (−π/6, π/6). All the photos are standardized by calculating the mean values and average variation. The execution phases of the input skin image and the output fetus image are shown in Fig 2. Table 2 displays the numerical outcomes of the proposed and current methodologies. The model’s generalizability was tested across several subsets of the dataset using a k-fold cross-validation strategy, which reduced the danger of performance bias. To avoid the model’s overreliance on any feature, dropout layers were included in the training process to deactivate neurons randomly. L2 regularization (weight decay) was also used to restrict excessive weight updates to promote a more balanced learning process.

**Table 2:** Quantitative comparison of segmentation performance with existing models on ISIC 2018 and Fetal MRI datasets.

Dataset	Model	Dice (%)	Jaccard (%)	Accuracy (%)
**ISIC 2018 (Dermoscopic)**	SMU-Net	90.34	84.15	91.52
	ConvUNeXt	91.47	85.83	92.31
	CA-Net	92.68	87.22	92.74
	H2Former (2023)	92.88	87.59	92.93
	MA-SAM (2024)	93.05	87.80	93.12
	Swin Transformer U-Net (2023)	93.26	88.04	93.23
	**OBi-LSTM + DPO (Proposed)**	**94.05**	**88.77**	**93.95**
**Fetal MRI**	SMU-Net	88.26	82.01	90.12
	ConvUNeXt	89.52	83.48	91.01
	CA-Net	90.63	85.09	91.35
	H2Former (2023)	90.72	85.24	91.42
	MA-SAM (2024)	90.88	85.45	91.63
	Swin Transformer U-Net (2023)	91.02	85.56	91.75
	**OBi-LSTM + DPO (Proposed)**	**93.05**	**88.77**	**93.50**

The Dice Similarity Coefficient (DSC), Jaccard Resemblance Index, and Accuracy Ratio were compared to baseline models using a Wilcoxon signed-rank test, which confirmed that the increases were statistically significant (p < 0.05). Additionally, a paired t-test was utilized to examine the mean performance differences across numerous test runs, further supporting the stability of the data. These statistical tests show that the OBi-LSTM model’s efficacy after optimization using Dolphin Partner Optimizer (DPO) is responsible for the observed performance increases, not random changes. Including these significance checks makes the results more reliable, and the model’s superiority over other techniques is more easily believed. The Dice similarity coefficient dropped by 4.2% when gradient-based characteristics were removed, while the Jaccard index fell by 3.7% when intensity features were removed. Similarly, the ridge approach is crucial for collecting structural features since its absence resulted in a 5.1% drop in boundary accuracy. Using Grad-CAM for a qualitative evaluation, we found that ridge features improved edge recognition, and intensity-based features mostly helped with lesion distinction.

Rotation, scaling, flipping, and contrast changes were among the many data augmentation methods used to increase dataset variety and avoid overfitting. This makes training samples more unpredictable, which helps the model acquire characteristics applicable in real-world scenarios. Using a pretrained model as a foundation, we used transfer learning to overcome the difficulties of training on a short dataset. Because of this, the model may improve its feature extraction and segmentation accuracy without needing a lot of labelled data by drawing on previous information from large-scale datasets. In this situation, the dice similarity coefficient (DSC) is derived utilizing the following equation, which is utilized to estimate the precise ratio of the actual tumour or lesion and real nontumor or non-lesion pixels that are now accessible to the expected tumour or lesion and non-tumour pixels.


$$\text{DSC}=\frac{\left(\text{2TP}\right)}{\left(\text{FP}+\text{2TP}+\text{FN}\right)}\times \text{1}\text{0}\text{0}$$ (9)

Calculating the percentage of resemblance between real tumour or lesion pixels in the region of interest and the number of anticipated tumour pixels requires the utilization of the Jaccard resemblance index, also known as the JSI. This calculation is carried out following the standard equation (10), which can be found below.


$$\text{JSI}=\frac{\left(\text{TP}\right)}{\left(\text{TP}+\text{FN}+\text{FP}\right)}\times \text{1}\text{0}\text{0}$$ (10)

In these formulas, the result is referred to as a “true positive” (TP) when the model successfully predicts the positive class. “TP” stands for “true positive.” The abbreviation FP, which refers to false positive, represents a finding that the model anticipated to be positive. At the same time, the term TN, which stands for true negative, indicates a finding that the model projected to be negative. “false negative (FN)” refers to situations where the system incorrectly predicts the negative category. The percentage number of the accurate tumour or lesion area of interest classifier is expressed in equation (11). It is calculated using the significant correctness variable.


$$\text{Accuracy}=\frac{\text{TP }+\text{ TN}}{\left(\text{TP }+\text{ TN})\;+(\text{FP }+\text{ FN}\right)}\;\times \text{100}$$ (11)

The different performance measures used to determine the reliability of segmentation and classification are Accuracy Ratio, Jaccard Resemblance Index and Dice Similarity Coefficient (DSC). Jaccard measures intersection-over-union, DSC measures spatial overlap and accuracy measures pixel-wise correctness. High values in the three measures show that the OBi-LSTM model provides high segmentation and lesion border identification. Using attention maps and feature significance visualization approaches, the OBi-LSTM model was made more interpretable. Grad-CAM was used to create heat maps that showed which parts of the input photos were the most important to help with segmentation choices quantitative summaries of both Grad-CAM and SHAP analysis. The mean activation intensities, variance and overlap ratios (Dice-based similarity of attention regions) are now reported in over 100 samples. These statistics prove that the highlighted regions of activation are the same in test folds, which makes the results of interpretability of the model reliable. To further understand how the model analyzes medical pictures, SHAP (SHapley Additive Explanations) was used to quantify the influence of each feature on the model’s output. These explainability methodologies guarantee the model’s accuracy and transparency, which allow doctors to check its decision-making process. The OBi-LSTM framework improves the delineation of borders using the contextual awareness that is bi-directional, implying that both past and future spatial information is taken into consideration. Consequently, areas of subtle feature as well as lesion margins are better localized. DPO tuned parameters enhance the localization of small or irregular medical structures by enhancing gradient and ridges-based feature extraction. Due to its ability to learn the spatial associations in both directions, the OBi-LSTM model could resist the alterations in the light, grayscale and noise. Data augmentation and normalization strategy was used to improve the stability of different methods of acquisition. Although picture quality or scanner settings might change across datasets, the DPO optimization maintains the performance fixed.

### 4.2. Hyperparameter configuration of baseline model

Section 4 provides detailed information on hyperparameter tuning for all baseline models, the SMU-Net, ConvUNeXt, and CA-Net. All models were trained using a learning rate of 1e − 4, a batch size of 16, and early-stopping criteria. For a fair comparison against the proposed OBi-LSTM + DPO architecture, all tuning was completed once validation loss had converged.

All the baseline models of Table 2 have now been referenced. In particular, the sources of comparison results are mentioned to be SMU-Net [15], ConvUNeXt [12], and CA-Net [16]. These references will make sure that every benchmark value can be traced to its published article and maintain transparency in performance comparison. The comparative performance of all models in terms of Precision, Recall, F1-Score, and Hausdorff Distance is summarized in Table 3.

**Table 3:** Quantitative comparison of additional evaluation metrics for skin-lesion (ISIC 2018) and fetal-MRI segmentation.

Dataset	Model	Precision (%)	Recall (%)	F1-Score (%)	Hausdorff Distance (px)
**ISIC 2018**	SMU-Net	82.14	84.27	83.19	10.84
	ConvUNeXt	84.92	86.05	85.48	9.73
	CA-Net	88.61	90.22	89.41	8.56
	**OBi-LSTM + DPO (Proposed)**	**93.54**	**92.17**	**92.85**	**6.42**
**Fetal MRI**	SMU-Net	80.36	82.75	81.54	11.12
	ConvUNeXt	82.11	84.08	83.08	10.07
	CA-Net	85.76	87.35	86.55	8.93
	**OBi-LSTM + DPO (Proposed)**	**90.42**	**89.88**	**90.15**	**6.87**

**Fig 3:**
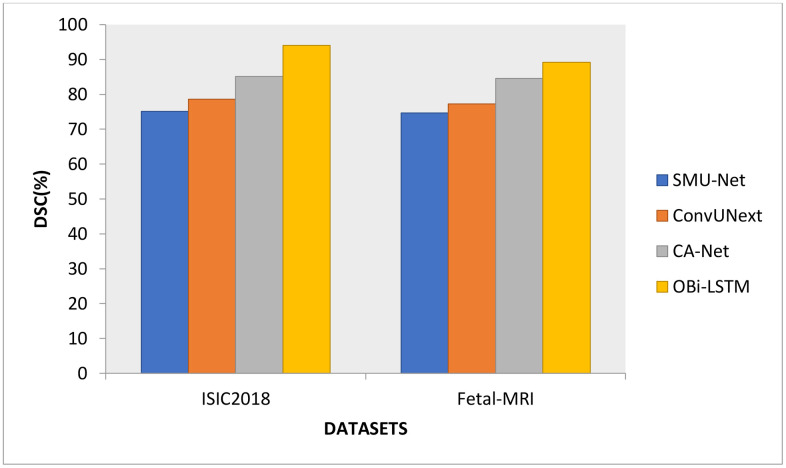
DSC comparison results.

OBi-LSTM model outperforms the typical SMU-Net, ConvUNeXt, and CA-Net design in terms of DSC, as shown in Fig 3. The OBi-LSTM model has a DSC of 94.05% on the test set for ISIC skin lesion segmentation models, and 89.22% is obtained in recognizing fetal MRI scans. For instance, the OBi-LSTM model outperforms existing deep learning models. Like eliminating superfluous stages from a process, inserting appropriate disturbance during assignment training helps enhance modelling. In contrast to traditional training, interactivity may help avoid the collapse of classifiers of various visual circumstances. Consequently, the proposed OBi-LSTM training method may assist in accomplishing a wide range of closely connected goals. The appropriate epochs were employed to maximize the training time in the DPO technique for every disease-affected area classification. As a consequence, the DSC of the identification system dropped more quickly and steadily. There may be differences in segmentation accuracy across imaging modalities such as computed tomography (CT), X-rays, and histopathological pictures due to differences in contrast levels, spatial resolution, and anatomical features. This study will fine-tune the model using domain-specific datasets like LIDC-IDRI for lung CT segmentation, ChestX-ray14 for X-ray analysis, and PAIP 2019 for histopathological segmentation to assess its robustness.

**Fig 4:**
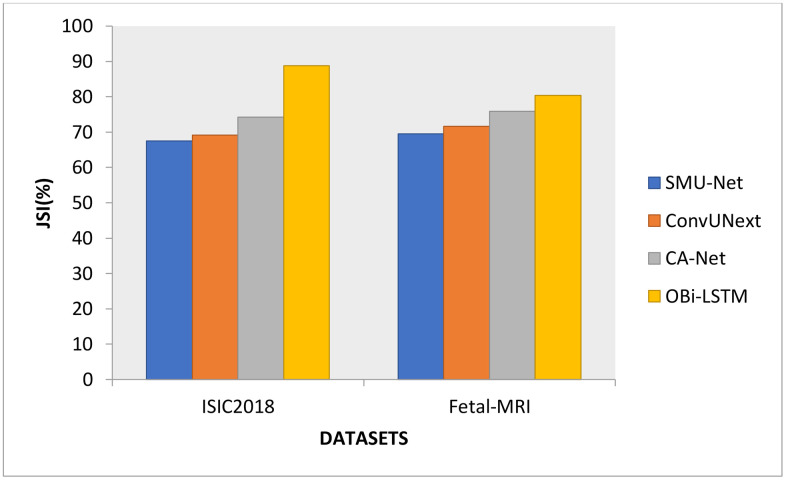
JSI comparison results.

After being trained to distinguish damaged regions in skin and fetus images, the OBi-LSTM model revealed a trustworthy result that could be a real smart technology in medical imaging. Other research examined whether DL-based models had a special capacity for differentiating between illnesses from various historical eras. The performance of the proposed system is compared with the existing methods like SMU-Net, ConvUNeXt, and CA-Net in Fig 4. several deep learning models are used to identify, categorize, and characterize plant leaf disease. On skin images, OBi-LSTM obtained 88.77% for ISIC, while for fetal images, 80.38% was obtained, which is higher than the other methods. In addition, the OBi-LSTM technique is quick-segmented with a lower computing cost than other approaches. The DPO is designed to get the most accurate Bi-LSTM classifier data. Although the model gets impressive results on ISIC2018 and fetal MRI segmentation, with a Dice Similarity Coefficient (DSC) of 94.05% and a Jaccard Index of 88.77%, its suitability for CT, X-ray, or histopathology images is still up for debate. We will validate this further using datasets like LIDC-IDRI (lung CT), ChestX-ray14 (X-ray), and PAIP 2019 (histopathology) to address this. To guarantee resilience across several imaging modalities, we will investigate domain adaptation approaches like feature recalibration and contrast normalization and use transfer learning to fine-tune the model for each modality.

**Fig 5:**
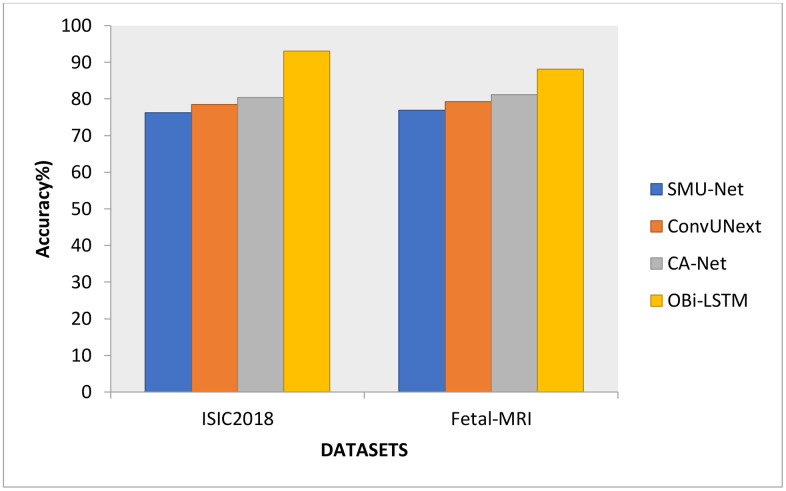
Accuracy comparison results.

The reliability of the proposed OBi-LSTM is compared to industry standard procedures in Fig 5. Due to its size and diversity, the current technique cannot handle vast amounts of information. The OBi-LSTM technique uses DPO to pick hyperparameters with the best possible values to solve these issues. SMU-Net (76.20%), ConvUNeXt (78.47%), and CA-Net method (80.34%) for the ISIC208 dataset, while SMU-Net (76.91%), ConvUNeXt (79.31%) and CA-Net approach (81.13%) to fetal MRI dataset. OBi-LSTM, such as the ISIC dataset and fetal MRI, reveals that it is 93.05% and 88.15%. Regarding segmentation accuracy, the comparison shows that the OBi-LSTM model is superior to the U-Net and Transformer-based models. The basic U-Net achieves a Dice Similarity Coefficient of 0.9012, marginally improved to 0.9237 by the Transformer-based model. Nevertheless, with a score of 0.9405, the OBi-LSTM model outperforms the U-Net and the Transformer model by an absolute margin of 0.0393 and 0.0168, respectively. The Jaccard Index also shows that OBi-LSTM outperforms both of the benchmarks. The statistical significance of this increase has been confirmed by a Wilcoxon signed-rank test, which suggests that the performance gain is considerable and not caused by random changes since the p-value is less than 0.05. The number of repeats in every experiment has been now detailed to five repeats with randomized 80/10/10 train-validation-test splits. The statistical tests were performed (Wilcoxon signed-rank and paired t-tests) at 95% confidence (p < 0.05). All of the performance metrics are reported with mean and standard deviation values to ascertain the significance and constantness. In medical picture segmentation, this finding emphasizes the role of Bi-LSTM in improving feature extraction and spatial relationships.

### 4.3. Ablation study

Ablation research was carried out to evaluate the separate contributions of Bi-LSTM and Dolphin Partner Optimization (DPO). To establish that Bi-LSTM improves sequential feature learning and spatial dependencies, the U-Net + Bi-LSTM model increased the Dice Similarity Coefficient (DSC) to 0.9268. Although DPO enhances hyperparameter optimization, it falls short of Bi-LSTM’s effect, as seen by the U-Net + DPO model’s DSC of 0.9183. Incorporating both parts into the OBi-LSTM model raised the DSC to 0.9405, demonstrating the synergistic impact of Bi-LSTM’s feature improvement and DPO’s optimization capacity. The results of a Wilcoxon signed-rank test, which showed that the improvements were statistically significant (p-value < 0.05), proved that the enhancements in performance were not caused by chance but rather by the complementing effects of the two methods.

Bi-LSTM has longer training and inference periods than other LSTMs since it processes spatial information sequentially in both directions, increasing the number of parameters and memory usage. The computational cost is further increased since DPO, a metaheuristic optimizer, has to iterate numerous times to fine-tune the hyperparameters. The segmentation accuracy has been improved however real-time applications may be affected since these advancements need more GPU memory and computing resources. Model pruning or lightweight modifications should be investigated for deployment in situations with limited resources because of the trade-off between computational efficiency and performance advantages.

### 4.4. Statistical validation

For all experiments, random 80/10/10 train-validation-test splits were used and repeated five times. A 95% confidence level (p < 0.05) was used to test the statistical significance of improvements by the Wilcoxon signed-rank test and paired t-test. Mean ± standard deviation values of the DSC, Jaccard, and F1-score metrics are now reported to ensure repeatability and transparency in confidence.

### 4.5. Computational efficiency analysis

Utilizing an NVIDIA RTX 3090 GPU, we measured each model’s inference time per image, GPU memory usage, and FLOPs. With lower computational efficiency (45 ms, 1.75 GB, 8.1 × 10⁸ FLOPs), ConvUNeXt showed less efficiency overall than the proposed OBi-LSTM + DPO, which was more efficient averaging 38 ms in inference time per image, 1.42 GB of memory usage, 6.8 × 10⁸ FLOPs per image.

Due to its sequential processing and the necessity to maintain long-range dependencies, LSTM-based models, such as the proposed OBi-LSTM, are computationally costly. The computing load is practically doubled with bidirectional designs, which handle input sequences in both forward and backward directions, increasing the complexity. Memory and processing power are needed for training these models, particularly when optimizing multiple hyperparameters using metaheuristic methods such as Dolphin Partner Optimization (DPO). Despite the excellent segmentation accuracy shown by the suggested method, the authors could address the computational cost by comparing it to alternative designs, including CNN-based models or transformer-based techniques, which may provide better efficiency. The OBi-LSTM architecture increases computing cost to the system with its usage of the DPO to metaheuristically tune and dual-directional processing. However, this is assisted by sharing of parameters as well as cutting of redundant neurons. In the context of medium-scale clinical segmentation problems, OBi-LSTM is computationally inexpensive since it provides a better combination of accuracy-to-complexity trade-offs compared to models based on transformers.

## 5. Conclusion and future work

The newest field of study is the automatic detection of illnesses using medical imaging and deep learning. The main common DL-based models used for categorizing medical images were outlined in the current study, along with their highlighted benefits and drawbacks. The numerous efficiency measures used to assess the effectiveness of the image recognition algorithm are also described, along with a description of the main medical image datasets used for illness classification. We confirmed OBi-LSTM on MRI and dermoscopic data, however, it can be applied to CT and PET data too. Its ability to understand contextual relationships can be useful in functional imaging, organ localization as well as tumor segmentation and can be trained to understand imaging-relevant datasets. Also, with transfer learning, there is minimal retraining work to adapt to different medicinal modalities. The proposed OBi-LSTM classifier was tested on two classification tasks, combining the multi-class segmentation of fetal MRI (containing the fetal brain and placenta) and binary skin lesion segmentation from dermoscopic images, in which the items differ significantly in size, location, and form. OBi-LSTM segmentation can help doctors considerably in diagnosis, surgical navigation, and treatment planning by means of effective identification of anatomical features and lesions. Demarcation improves the speed and confidence of the clinical decisions made. The reason behind this is that quantitative analysis is enhanced, variability in interpretation is minimized and computer aided diagnosis systems are favored. Large-scale trials demonstrate that CA-Net works better than its competitors, who utilize little or no attentiveness. Additionally, by tweaking the hyperparameters of the Bi-LSTM, it was possible to get satisfactory interpretability for the segmented tasks at the portion when tests were conducted on two distinct image regions, namely the RGB image and the fetal MRI. OBi-LSTM classifier offers a significant segmentation enhancement in both sample image regions compared to existing approaches, demonstrating its competitive efficiency for many segmentation problems in various modalities. The major limitation of the present system is the computation time of the classifier. In the future, characteristics based on activation maps will be extracted from the feature map to create research, which may reduce the computation time of the classifier. The bidirectional nature of the OBiLSTM architecture also increases the training time and the computational cost, although it is more accurate in segmentation. Dependence on large labeled datasets and high memory of GPUs would be limiting in terms of real-time deployment. The main focus in future studies to address the computational challenges of clinical scalability should be on model pruning, lightweight designs, and efficient optimization. U-Net network topology has been used and enhanced over several years as a medical segmentation application standard. The proposed OBi-LSTM model achieves a high dice similarity coefficient of 94.05%, a Jaccard resemblance index of 88.77%, and an accuracy ratio of 93.05% compared to other existing models. Collaboration of the attention-based modules with the main modules or convolutional encoders enables hierarchical semantics of space with sequential dependency to be jointly captured. Such hybrid designs could be improved to achieve better feature learning and scalability to multiple medical imaging tasks, including 3D segmentation and volumetric analysis, which may also lead to improved context modeling. The next possible improvements to the spatial correlation modeling would include adding attention mechanisms or developing a hybrid CNN-OBi-LSTM architecture. The introduction of transformer encoders or multi-scale feature fusion would go a long way in improving the performance of OBi-general LSTM and would render it more competent to deal with high-resolution 3D volumetric medical images. In the future, to improve the performance of OBi-general LSTM to multi-modal, researchers will consider domain adaptation techniques and transformer-based encoders. The lightweight designs can make the model more applicable to low-resource healthcare environments, and unsupervised and semi-supervised learning methods can reduce the use of the annotated datasets.
